# MiR-375 Has Contrasting Effects on Newcastle Disease Virus Growth Depending on the Target Gene : Erratum

**DOI:** 10.7150/ijbs.89210

**Published:** 2023-11-09

**Authors:** Xinglong Wang, Yanqing Jia, Xiangwei Wang, Chongyang Wang, Changjie Lv, Xiaoqin Li, Zhili Chu, Qingsong Han, Sa Xiao, Shuxia Zhang, Zengqi Yang

**Affiliations:** College of Veterinary Medicine, Northwest A&F University, Yangling, Shaanxi Province 712100, PR China

In our paper, the author noticed an error in Figure 8C. There was a non-subjective typographical error of the Sponge-NC in the Figure 8C, which was same as Figure 7C. We checked the original data again and made sure that the conclusion of the article was not affected by the error. In this regard, all authors have agreed to the erratum, and we apologize for any inconvenience caused by the negligence in our work.

Figure 8C should be corrected as follows.

## Figures and Tables

**Figure 8:**
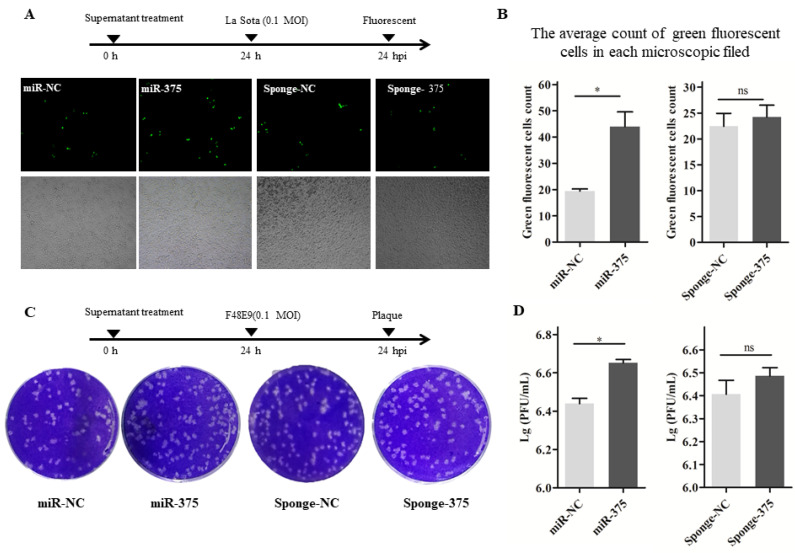
Ccorrect image.

